# WITHIN- AND BETWEEN-COUNTRY DIFFERENCES IN DAILY ASSOCIATION OF VIEWS OF AGING AND NEGATIVE AFFECT

**DOI:** 10.1093/geroni/igad104.1538

**Published:** 2023-12-21

**Authors:** Amit Shrira, Yuval Palgi, Shevaun Neupert

**Affiliations:** Bar Ilan University, Ramat Gan, HaMerkaz, Israel; University of Haifa, Haifa, Hefa, Israel; North Carolina State University, Raleigh, North Carolina, United States

## Abstract

Few studies found cultural differences, both across and within countries, in the association between subjective views of aging (VoA) and psychological concomitants. However, a question remains whether such cultural differences exist on a daily level. We therefore examined cultural differences in daily covariation of VoA and negative affect (NA) within an Israeli sample (comparing Israeli Jews to Arabs) and between countries (comparing the Israeli sample to an American sample). The Israeli sample included 75 participants (age ranged 50 to 88, 36% Arabs). The American sample included 77 participants (age ranged 50 to 82). Participants reported subjective age, subjective accelerated aging, ageist attitudes, and NA across 14 days. Within-person variability ranged from 28% (ageist attitudes) to 52% (NA) of the total variance in the Israeli sample, and 26% (subjective age) to 38% (subjective accelerated aging) in the American sample. On days respondents felt younger, felt to be aging slower, or had less ageist attitudes, they also reported lower NA. Cross-level interactions showed that the associations of subjective age and subjective accelerated aging with NA were significantly stronger among Israeli Arabs (versus Israeli Jews) and significantly stronger in the Israeli sample (versus the American sample). The daily covariance between ageist attitudes and NA remained similar across cultural groups. These findings attest to the importance of taking a cultural perspective on daily relationships between VoA and psychological concomitants. Our talk will discuss potential explanations for the abovementioned cultural differences and similarities and will point to future directions in our Subjective AGES project.

